# Reproductive history and blood cell DNA methylation later in life: the Young Finns Study

**DOI:** 10.1186/s13148-021-01215-1

**Published:** 2021-12-20

**Authors:** Emily W. Harville, Pashupati P. Mishra, Mika Kähönen, Emma Raitoharju, Saara Marttila, Olli Raitakari, Terho Lehtimäki

**Affiliations:** 1 Department of Epidemiology, Tulane University School of Public Health and Tropical Medicine, New Orleans, LA 70112 USA; 2 Department of Clinical Chemistry, Faculty of Medicine and Health Technology, Tampere University, 33520 Tampere, Finland; 3 Finnish Cardiovascular Research Center - Tampere, Faculty of Medicine and Health Technology, Tampere University, 33521 Tampere, Finland; 4 Department of Clinical Chemistry, Fimlab Laboratories, Tampere, Finland; 5 Department of Clinical Physiology, Tampere University Hospital, and Finnish Cardiovascular Research Center - Tampere, Faculty of Medicine and Health Technology, Tampere University, 33521 Tampere, Finland; 6 Department of Molecular Epidemiology, Faculty of Medicine and Health Technology, Tampere University, Tampere, Finland; 7 Gerontology Research Center, Tampere University, Tampere, Finland; 8 Research Center of Applied and Preventive Cardiovascular Medicine, University of Turku, Turku, Finland

**Keywords:** DNA methylation, Epigenetics, Pregnancy, Reproductive history, Pre-eclampsia

## Abstract

**Background:**

Women with a history of complications of pregnancy, including hypertensive disorders, gestational diabetes or an infant fetal growth restriction or preterm birth, are at higher risk for cardiovascular disease later in life. We aimed to examine differences in maternal DNA methylation following pregnancy complications.

**Methods:**

Data on women participating in the Young Finns study (n = 836) were linked to the national birth registry. DNA methylation in whole blood was assessed using the Infinium Methylation EPIC BeadChip. Epigenome-wide analysis was conducted on differential CpG methylation at 850 K sites. Reproductive history was also modeled as a predictor of four epigenetic age indices.

**Results:**

Fourteen significant differentially methylated sites were found associated with both history of pre-eclampsia and overall hypertensive disorders of pregnancy. No associations were found between reproductive history and any epigenetic age acceleration measure.

**Conclusions:**

Differences in epigenetic methylation profiles could represent pre-existing risk factors, or changes that occurred as a result of experiencing these complications.

**Supplementary Information:**

The online version contains supplementary material available at 10.1186/s13148-021-01215-1.

## Background

Pregnancy is a window to later-life health. Pregnancy complications such as gestational diabetes, hypertensive disorders (gestational hypertension and pre-eclampsia), fetal growth restriction (measured as small-for-gestational-age or low birthweight), and preterm birth predict cardiovascular morbidity and mortality [1–3]. Higher parity is also associated with cardiovascular disease risk [4]. Other body systems, including the immune and musculoskeletal systems, also show long-term alterations after pregnancy [5, 6]. In addition, it has been suggested that reproductive life history contributes to aging. Very high parity and adolescent pregnancy have been associated with increased disability [7, 8], worse physical role functioning [9, 10] and physical decline [10, 11].

It is unclear whether pregnancy complications reveal a pre-existing high baseline, or induce changes that lead to later disease [12]. The physiologic demands of pregnancy may merely act as a stress test, revealing underlying predispositions for vascular and endothelial dysfunction or unmasking sub-clinical disease [13]. On the other hand, it is possible that pregnancy complications reprogram the cardiovascular system and put women on a trajectory for development of disease [14]. One mechanism by which pregnancy complications could contribute to later-life health is by altering epigenetic markers that affect cardiovascular function. Epigenetic markers, particularly changes or differences in DNA methylation, are associated with cardiometabolic outcomes [15], lipoprotein metabolism [16], and subclinical atherosclerosis, i.e., carotid intima-media thickness [17]. Such changes in methylation may persist after exposure to a risk factor and lead to changes in gene expression that contribute to the progression of cardiovascular disease over time.

Recently, combined sets of epigenetic markers have been developed to measure aging [18, 19], but only a few studies have linked these epigenetic clocks with reproductive history. A study of young Filipina women (n = 397) found that methylation age (measured with the Horvath calculation) increased with gravidity [20], and among white non-Hispanic U.S. women in the Sister Study (n = 2356), there was a small association between number of births and epigenetic age, measured with three epigenetic clocks [21]. A small study indicated that pregnancy to postpartum was associated with a reduction in epigenetic age [22]. The Sister Study also found that women with a history of abnormal glucose tolerance during pregnancy had increased epigenetic age by the Hannum and Levine clocks [21], though no associations were found with hypertensive disorders of pregnancy. Maternal epigenetic age was also found to be associated with shorter gestational length and lower birthweight in one study of 77 diverse California women [23].

While epigenetic studies have found associations between experiencing pregnancy complications in utero and methylation in placental, cord blood, and childhood tissue samples [24], few studies have examined later-life methylation in the mother, which could indicate possible long-term effects of these complications on health. We examined whether history of pregnancy complications was associated with whole blood DNA methylation later in life, using an epigenome-wide association study in the Young Finns study.

## Methods

### Study population

The methods for the Young Finns study have been described in detail previously [25]. Briefly, 3596 children and adolescents (1832 girls) aged 3–18 years, randomly chosen from population registers, were enrolled in 1980. Follow-up assessments were conducted every three years between 1980 and 1992, and then in 2001 and 2007. Retention rates for women were 81% in 1983, 72% in 1986, 68% for clinical examinations in 2001, and 66% for 2007. There was no difference in baseline cholesterol, blood pressure, or BMI between those lost to follow-up and those continuing to participate [25].

### Reproductive history

Data from the cardiovascular study cohort were merged with the Finnish national birth registry. The birth registry contains information on every birth in Finland since 1987, including data on the pregnancy. Of the 1832 women originally enrolled in the Young Finns study, 1316 had at least one singleton birth in the registry.

Reproductive outcomes were largely considered in three areas: parity, birth outcomes, and pregnancy complications. Parity was assessed as the number of births in the registry. The birth registry data include direct reports of some medical information, as well as up to 10 spaces to record diagnoses using ICD-9 and ICD-10 codes. Pre-eclampsia was defined as a recorded diagnosis of eclampsia (n = 1) as well as ICD-9 codes 6424–6427 and ICD-10 codes O11, O12, O14, and O15. Overall hypertensive disorders of pregnancy were defined as any hypertensive disorder specified as having begun during pregnancy (ICD-9 codes 6423–6427, ICD-10 codes O12-O15). Gestational diabetes (GDM) was defined as failing a glucose tolerance test, ICD-9 code 6488, or ICD-10 code O24. Preterm birth was defined as birth at < 37 weeks’ gestation, low birthweight as birthweight < 2500 g, and small-for-gestational-age as weight below the 10th percentile for gestational age, with percentiles based on this study population.

### Epigenetic analysis

#### DNA Methylation profiling and pre-processing

Whole blood (maternal) DNA of the YFS cohort (n = 1529 total from year 2011–12; 836 were included in the analysis of parity and 638 of pregnancy complications. Mean time between first birth and epigenetic measure 13.9 years; mean time between last birth and epigenetic measure 9.4 years) was obtained from EDTA-blood samples using a Wizard® Genomic DNA Purification Kit (Promega Corporation, Madison, WI, USA) according to the manufacturer’s instructions. Genome-wide DNA methylation levels were obtained using Infinium MethylationEPIC array according to the protocol by Illumina. All the analyzed samples have sum of detection *p*-values across all the probes less than 0.01. Logged (log2) median of methylated and unmethylated intensities of the analyzed samples clustered well visually. Further, samples for which reported sex did not match the predicted sex were excluded (n = 2). Background subtraction and dye-bias normalization was performed via noob method [26] followed by stratified quantile normalization. Probes with detection *p*-value more than 0.01 in 99% of the samples were filtered out. All pre-processing steps were performed using functions implemented in *minfi* R/Bioconductor package [27]. Batch effects were controlled by including the first 30 control probe-based principal components[28]; we also excluded cross-reactive probes and probes with SNPs [29, 30].

### Statistical analysis

Outcomes for all pregnancies prior to the visit with the epigenetic measure were combined for a summary variable indicating history of that complication. Multiple linear regression analysis with methylation beta values as outcome and reproductive factors as predictor was performed in R statistical software (v.3.5.1) [31]. The analysis was adjusted with parity, age, sex, body mass index (BMI), smoking, whole blood cell proportions estimated with Houseman method [32] and the first 30 principal components of control probes to control for technical covariates. We used a genome-level threshold (*p* < 5 × 10^−8^) as the criterion for significance. We also examined whether the differentially methylated CpG sites were enriched or depleted from specific genomic regions related to CpG islands or genes using a hypergeometric test with the *phyper* function in R. Annotation of CpG sites was based on information provided by Illumina. Biological pathways associated with the significant CpG sites were tested using Gene Ontology categories (GO) as well as Kyoto Encyclopedia of Genes and Genomes (KEGG) pathways with a test based on Wallenius’ noncentral hypergeometric distribution implemented in *missMethyl* R package [33].

Several indices of methylation age have been developed to reflect overall aging. Methylation age was modeled as an outcome with parity and history of pregnancy complications as predictors. We examined the Hannum [34], Horvath [18], PhenoAge [35], and GrimAge [36] age acceleration (residual difference of chronological and epigenetic age), all of which were calculated according to published methods. We additionally controlled for age at first birth, age at most recent birth, BMI in 2001, and parity (for analyses other than parity). All covariates were chosen as factors with strong biological relationships with reproductive complications.

The Young Finns study was approved by the local ethical committees (1st Ethical Committee of the Hospital District of Southwest Finland, Regional Ethics Committee of the Expert Responsibility area of Tampere University Hospital, Helsinki University Hospital Ethical Committee of Medicine, The Research Ethics Committee of the Northern Savo Hospital District and Ethics Committee of the Northern Ostrobothnia Hospital District) of the participating sites (ETMK: 88/180/2010).

## Results

The study population is described in Table 1. Approximately 20% (165) of women had no births recorded in the registry, while 20% (166) had one birth, and 60% (505) had two or more. One hundred and two (13%) were smokers in 2011.

**Table 1:** Women with methylation data in the Cardiovascular Risk in Young Finns Study

	N	%
Parity		
0	165	19.7
1	166	19.9
2 +	505	60.4
Smoking in 2011	102/798	12.8
Preterm birth	63/671	9.4
Low birthweight	36/671	5.4
Hypertensive disorder	63/671	9.4
Pre-eclampsia	9/671	1.3
Gestational diabetes	50/671	7.5
	Mean	SD
Age at epigenetic measure	41.4	5.0
Age at first birth	27.5	5.0
BMI at age 18*	21.8	2.9
BMI at epigenetic measure	26.5	5.1

^*^Visit nearest to age 18

No significant associations were found between methylation of any CpG sites and parity or preterm birth (Additional file 1: Fig S1). A history of low birthweight was associated with alterations in cg26757524 (beta − 0.01457, SE 0.0226, *p* = 3.83 × 10^−8^), within coding region of gene APPL2 in chromosome 12, a gene related to the cell proliferation, immune response, and cell metabolism. No other sites were found to be significantly associated.

History of pre-eclampsia was associated with both hyper- and hypomethylation at several sites (n = 206) (Table 2, Additional file 1: Table S1), as was history of gestational hypertension (Table 3, n = 31, Additional file 1: Table S2). Fourteen CpG sites met the significance criterion for both outcomes (starred in Tables 2 and 3): cg16202187 in HAUS8 (chromosome 19, protein coding gene), cg05583353 in AC092652.1 (chromosome 2), cg10918328 in LINC00391 (chromosome 13, RNA gene), cg24141991 in RNF26 (chromosome 11, protein coding—endosome organization), cg14453326 in DDX55 (chromosome 12, protein coding—RNA helicases), cg13709211 in MEG3 (chromosome 14, RNA gene), cg23834489 PSMD3 (chromosome 17, protein coding), and five sites not associated with a coding region (cg26460602 and cg08112740, chromosome 19; cg05223360, chromosome 1; cg05080811, chromosome 22; cg17324671, chromosome 2). Lambda was very close to 1, which suggests lack of inflation and no more false positives than expected (Additional file 1: Fig S2). No biological pathways met the criteria for significance (Additional file 1: Table S3). For pre-eclampsia, the significant hits were depleted from CpG island shores (*p* = 0.007). Among the gene regions, there was enrichment of significant hits in the gene body (*p* = 0.02) and also enrichment in TSS200 (200 nucleotides upstream of transcription start site, *p* = 0.03), but depletion in TSS1500 (200–1500 nucleotides upstream from TSS, *p* = 0.03). For pregnancy-induced hypertension, there was no enrichment/depletion detected, probably due to the very small number of significant hits.

**Table 2:** CpG sites with differential methylation in women with a history of pre-eclampsia. Top 20 most significant CpG sites. CpG sites that met the genome-wide significance criterion (*p* < 5 × 10^−8^) for both pre-eclampsia and gestational hypertension (starred*)

CpG	Beta	SE	*p*-value	Chromosome	Gencode basic name
cg16202187*	0.021	0.002	5.75 E − 25	chr19	HAUS8
cg05583353*	− 0.048	0.005	1.02 E − 22	chr2	AC092652.1
cg10918328*	0.042	0.004	1.16 E − 21	chr13	LINC00391
cg26460602*	− 0.059	0.006	1.39 E − 18	chr19	
cg03942922	− 0.054	0.006	5.81 E − 18	chr6	C6orf130
cg24936032	0.059	0.007	6.31 E − 18	chr5	HINT1
cg24141991*	− 0.045	0.005	1.48 E − 17	chr11	RNF26;RP11-334E6.10
cg23567310	− 0.058	0.007	2.47 E − 17	chr3	YEATS2-AS1
cg11835785	− 0.062	0.007	8.79 E − 17	chr19	KDM4B
cg08112740*	− 0.077	0.009	1.13 E − 16	chr19	
cg07904329	0.054	0.006	1.57 E − 16	chr11	
cg14453326*	0.044	0.005	1.89 E − 16	chr12	DDX55
cg13709211*	− 0.093	0.011	2.15 E − 16	chr14	MEG3
cg19570702*	− 0.069	0.008	2.23 E − 16	chr6	HCG25; VPS52
cg25273619	− 0.047	0.006	2.45 E − 16	chr13	DCUN1D2-AS2
cg05223360*	− 0.055	0.007	2.77 E − 16	chr1	
cg00263326	− 0.050	0.006	4.44 E − 16	chr7	AC007551.3
ch.15.33220631R	0.049	0.006	4.85 E − 16	chr15	RP11-323I15.5
cg16505237	0.056	0.007	5.17 E − 16	chr8	DGAT1
cg00926164	− 0.039	0.005	1.45 E − 15	chr19

**Table 3:** CpG sites differentially methylated in women with a history of hypertensive disorders of pregnancy. Top 20 most significant CpG sites. Sites that met the genome-wide significance criterion (*p* < 5 × 10^−8^) for both pre-eclampsia and gestational hypertension (starred*)

CpG	Beta	SE	*p* value	chr	Gencode Basic
cg20089264	0.0171	0.003	6.25 E − 11	chr22	ASCC2
cg16202187*	0.009	0.001	1.68 E − 10	chr19	HAUS8
cg14862827	0.011	0.002	1.79 E − 10	chr9	
cg09020375	− 0.038	0.006	7.65 E − 10	chr4	ART3
cg23694630	− 0.039	0.006	9.20 E − 10	chr10	
cg13081185	− 0.034	0.006	1.60 E − 09	chr11	SIK3
cg12522722	− 0.047	0.008	1.63 E − 09	chr11	ZW10;RP11-661I21.2
cg10918328*	0.018	0.003	2.64 E − 09	chr13	LINC00391
cg02068524	0.026	0.004	2.79 E − 09	chr4	
cg08687948	− 0.027	0.005	3.09 E − 09	chr7	CYTH3;CYTH3
cg06853492	− 0.025	0.004	5.69 E − 09	chr2	RAPGEF4
cg17324671*	− 0.032	0.005	5.92 E − 09	chr2	
cg26460602*	− 0.027	0.005	6.75 E − 09	chr19	
cg05223360*	− 0.027	0.005	6.86 E − 09	chr1	
cg15400652	− 0.019	0.003	8.20 E - 09	chr19	PSG2
cg09138430	0.016	0.003	1.04 E − 08	chr7	DBNL
cg23834489*	− 0.023	0.004	1.04 E − 08	chr17	PSMD3
cg04806794	0.028	0.005	1.11 E − 08	chr9	TLE4
cg18304195	0.026	0.004	1.40E − 08	chr3	KBTBD8
cg13709211*	− 0.044	0.008	1.92E − 08	chr14	MEG3

Pre-eclampsia is strongly associated with later-life blood pressure, and (oddly) inversely associated with smoking [37]. We examined previous analyses of DNA methylation for high blood pressure [38] and smoking [39] in this cohort, and did not identify any overlapping differentially methylated sites (Additional file 1: Table S4).

No methylation sites were associated with GDM at a high enough level to meet the significance criterion; the ten sites most strongly associated are listed in Table 4. No associations were found between parity or history of pregnancy complications and any of the epigenetic age acceleration measures examined (Table 5).

**Table 4:** Methylation sites most strongly associated with history of gestational diabetes. None meet the genome-wide significance criterion (*p* < 5 × 10^−8^)

CpG	Beta	SE	*p* value	chr	Gencode Basic name
cg19078878	− 0.031	0.006	1.44E − 07	chr6	
cg09150251	− 0.014	0.003	8.18E − 07	chr10	RP1-251M9.2
cg02260571	− 0.010	0.002	8.35E − 07	chr2	CCDC141
cg07519259	− 0.015	0.003	9.65E − 07	chr2	
cg07752304	0.004	0.001	1.12E − 06	chr19	ARID3A
cg13264059	0.007	0.001	1.62E − 06	chr10	PTPRE
cg00854172	− 0.033	0.007	2.22E − 06	chr2	AC011239.1;KLHL29
cg03987506	0.001	0.002	2.70E − 06	chr3	OXTR;CAV3
cg09228048	0.005	0.001	3.42E − 06	chr3	AC018816.4
cg11230062	− 0.018	0.004	3.44E − 06	chr1	U3
cg01150646	− 0.016	0.003	3.58E − 06	chr17	
cg10556782	− 0.010	0.002	3.77E − 06	chr19	ZNF266
cg11168533	− 0.015	0.003	4.43E − 06	chr9	GNE
cg11227068	− 0.017	0.004	4.46E − 06	chr20	ROMO1; NFS1
cg19284306	− 0.022	0.005	6.00E − 06	chr1	RP1-158P9.1
cg00958475	− 0.016	0.004	6.23E − 06	chr4	
cg03562652	0.010	0.002	7.40E − 06	chr2	CCDC74A
cg05164570	0.019	0.004	7.72E − 06	chr5	HARS; HARS2
cg16068780	− 0.020	0.004	8.43E − 06	chr12	SBNO1
cg10937341	− 0.011	0.002	9.63E − 06	chr9	UBE2R2

**Table 5:** Methylation age acceleration and pregnancy history before 2011 (n = 896)

	Age acceleration residual — Horvath			Age acceleration residual — Hannum			Age acceleration — PhenoAge			Age acceleration — Grimage		
Parity	beta	SE	*p*	beta	SE	*p*	beta	SE	*p*	beta	SE	*p*
0			0.31			0.70	0.00		0.68	0.00		0.36
1	− 0.93	0.53		− 0.20	0.45		0.51	0.65		− 0.14	0.41	
2	− 0.27	0.46		0.25	0.39		0.67	0.56		− 0.40	0.35	
3 +	− 0.12	0.51		0.17	0.43		0.31	0.61		− 0.63	0.39	
History of low birthweight	0.19	0.86	0.83	− 1.11	0.73	0.13	1.58	1.00	0.12	0.91	0.60	0.13
History of preterm birth	0.10	0.65	0.87	− 0.26	0.55	0.64	1.06	0.76	0.16	− 0.34	0.46	0.46
History of any hypertensive disorder	− 0.94	0.70	0.18	− 0.21	0.59	0.73	− 0.98	0.81	0.23	− 0.53	0.49	0.28
History of pregnancy-induced hypertension	− 0.57	1.08	0.60	− 0.63	0.92	0.49	0.11	1.26	0.93	− 1.03	0.76	0.18
History of pre-eclampsia	− 0.01	1.48	0.99	− 0.49	1.26	0.70	1.28	1.73	0.46	− 0.14	1.04	0.89
History of gestational diabetes	0.39	0.77	0.62	0.05	0.65	0.94	0.08	0.90	0.93	0.44	0.54	0.42
History of miscarriage	− 0.34	0.50	0.50	0.31	0.42	0.47	0.71	0.58	0.22	0.03	0.35	0.94

## Discussion

This study examined how history of pregnancy and pregnancy complications was associated with differences in whole blood DNA methylation later in life. We found no effects of parity on DNA methylation, while a single site was associated with a history of giving birth to a low birthweight child, and several sites were identified for pre-eclampsia and gestational hypertension. No associations were found with epigenetic age.

A single site was associated with a history of giving birth to a low birthweight child, cg26757524, within coding region of gene APPL2 in chromosome 12, a gene related to the cell proliferation, immune response, and cell metabolism. APPL2 expression has been related to adiponectin and thus to obesity, insulin metabolism, and diabetes [40, 41]. Methylation of several sites was associated with a history of hypertensive disorders of pregnancy, although there were no clear associations with specific biological pathways. The fourteen sites common to pre-eclampsia and pregnancy-induced hypertension were also not in genes previously associated with pre-eclampsia or pregnancy-induced hypertension, nor with related conditions like hypertension. Some of the genes were protein coding genes and have occasionally been associated with cancers [42–45]. However, the distribution of the significant CpGs in regard to CpG islands and genes differed from what would be expected by chance. CpG sites associated with pre-eclampsia were enriched in CpG islands, as has previously been reported for differentially methylated regions (DMRs) associated with pre-eclampsia in cord blood [46]. In line with this, as CpG islands often overlap transcription start sites (TSSs), we also identified enrichment in TSS200 (200 nucleotides upstream of transcription start site). Reproductive history is a strong predictor of cardiovascular health [4], and cardiovascular health associated with epigenetic markers [47], so it is somewhat surprising there were not more associations found. This suggests pregnancy-related complications may have their effects on later blood pressure through other pathways.

In previous epigenetic research, pre-eclampsia is the most extensively studied of the pregnancy complications, although more often methylation of the placenta rather than blood. A few studies have targeted specific hypotheses: pre-eclampsia has been associated with hypermethylation of HLA-G promoter (chromosome 6p22.1) [48], hypomethylation of the CTGF promoter, differential methylation of the genes regulating placental nitric oxide (DDAH2) [49], and hypermethylation of 5,10-MTHFR [50]. Although none of these were clearly differentially methylated in this analysis, one site in the MTHFR gene was nominally associated with history of pre-eclampsia (beta 0.0078, SD 0.002, *p* = 0.0003). Comparing this study with an analysis of monozygotic twin study comparing sisters, one who had a complicated pregnancy and one who did not [51], also failed to establish any points of overlap. A previous untargeted, whole genome methylation sequencing of a family with multiple cases of pre-eclampsia identified DMRs in several genes [52]. None of the regions identified in that project were identified by the analytic strategy here; however, methylation of three overlapped regions in the TGFB3, DLX5, and LRP1B regions that were nominally significant in this analysis.

No epigenetic sites were strongly enough associated with GDM to meet the significance threshold, which may be due in part to the limited number of cases and corresponding lower power. A few previous studies have examined associations between peripheral blood methylation (measured during pregnancy) and GDM [53–58]. With the exception of a single CpG site that was nominally significant in both our analysis and the Enquobahrie analysis (in the DDR1 gene), no overlap between these studies’ results and ours was found.

Only a few studies have previously considered the relationship between maternal epigenetic markers and other reproductive outcomes. One study found substantial differences in methylation between pregnant and non-pregnant women, but not between postpartum and nulligravid women [59], suggesting that epigenetic changes related to pregnancy are transient, consistent with our finding of lack of association with parity. Another found that women became younger, epigenetically, with pregnancy [22]. One area of inquiry is the extent to which complications such as pre-eclampsia are driven by the fetal, rather than maternal, genome and epigenome [60]. Birth of twins alters methylation in children of subsequent births: two siblings born after a twin birth have methylation patterns more similar than those born before, or before and after, such a birth [61].

Although there is evidence for parity, hypertensive disorders, and gestational diabetes being associated with biological markers of aging [9, 10, 62], we found no association between reproductive history and epigenetic age acceleration, in contrast to some previous studies [20, 21]. This may be due to the relatively low parity in our population. We concur with these studies in finding no association between hypertensive disorders and epigenetic age.

Strengths of the analysis include the well-characterized cohort and essentially complete linkage for pregnancies. Limitations of the study include the variable length of time between the complications and the methylation measurement, the limited sample size for many complications, and the lack of a replication cohort. This study found no effect of pregnancy per se on methylation (either number of pregnancies or nulliparous/parous), which may be of importance in study design: if pregnancy truly has no effect, epigenetic measures taken postpartum can be used to investigate maternal methylation effects in pregnancy. However, this finding needs to be replicated in larger cohorts, and in studies with measures before and after pregnancy. Other methods exist for pipeline analysis, such as ENmix [63] but this analysis incorporates standard, widely used methods.

## Conclusions

Epigenetic changes are one way that pregnancy complications could exert effects on later-life health. Future studies should incorporate measures before and after pregnancy, to determine how pregnancy might alter or reveal cardiovascular risk. By determining whether this epigenetic predisposition is present prior to pregnancy or only later, we may identify timing for interventions. Such analysis is particularly important for pre-eclampsia, which seems to have multiple effects across systems and for later-life health, and for gestational diabetes, with its strong relationship with diabetes risk. One important question will be whether these complications affect later-life health by the same biological pathways as for people who develop these illnesses with no history of pregnancy complications, or whether they represent a separate pathway. By establishing the biological pathways, we can determine how these complications change biological pathways that affect lifelong health. In addition, the interaction of these biological factors with social ones should be examined. Putting together these factors will be important for understanding how chronic disease develops in women.

## Supplementary Information

**Additional file 1.** Supplementary tables and figures.

## Data Availability

The dataset supporting the conclusions of this article were obtained from the Cardiovascular Risk in Young Finns Study (YFS) which comprises health-related participant data. The use of data is restricted under the regulations on professional secrecy (Act on the Openness of Government Activities, 612/1999) and on sensitive personal data (Personal Data Act, 523/1999, implementing the EU data protection directive 95/46/EC). Due to these restrictions, the data cannot be stored in public repositories or otherwise made publicly available. Data access may be permitted on a case by case basis upon request only. Data sharing outside the group is done in collaboration with the YFS group and requires a data-sharing agreement. Investigators can submit an expression of interest to the chairman of the publication committee (Prof Mika Kähönen, Tampere University, Finland) and the responsible investigator of Genetic and Epigenetic part of YFS (Prof Terho Lehtimäki, Tampere University, Finland).

## Abbreviations

GDM: Gestational diabetes mellitus
YFS: Young Finns Study
DMR: Differentially methylated region
SNP: Single nucleotide polymorphism

## References

[1] Cirillo PM, Cohn BA (2015). Pregnancy complications and cardiovascular disease death: 50-year follow-up of the Child Health and Development Studies pregnancy cohort. Circulation.

[2] Bohrer J, Ehrenthal DB (2015). Other adverse pregnancy outcomes and future chronic disease. Semin Perinatol.

[3] Lind JM, Hennessy A, McLean M (2014). Cardiovascular disease in women: the significance of hypertension and gestational diabetes during pregnancy. Curr Opin Cardiol.

[4] Rich-Edwards JW, Fraser A, Lawlor DA, Catov JM (2014). Pregnancy characteristics and women's future cardiovascular health: an underused opportunity to improve women's health?. Epidemiol Rev.

[5] Smith MD, Russell A, Hodges PW (2008). Is there a relationship between parity, pregnancy, back pain and incontinence?. Int Urogynecol J Pelvic Floor Dysfunct.

[6] Rivara AC, Miller EM: Pregnancy and immune stimulation: re-imagining the fetus as parasite to understand age-related immune system changes in US women. Am J Hum Biol 2017, 29(6). 10.1002/ajhb.23041 28712140

[7] Akin B, Ege E, Kocoglu D, Arslan SY, Bilgili N (2010). Reproductive history, socioeconomic status and disability in the women aged 65 years or older in Turkey. Arch Gerontol Geriatr.

[8] Hank K (2010). Childbearing history, later-life health, and mortality in Germany. Popul Stud (Camb).

[9] Kington R, Lillard L, Rogowski J (1997). Reproductive history, socioeconomic status, and self-reported health status of women aged 50 years or older. Am J Public Health.

[10] Camara SM, Pirkle C, Moreira MA, Vieira MC, Vafaei A, Maciel AC (2015). Early maternal age and multiparity are associated to poor physical performance in middle-aged women from Northeast Brazil: a cross-sectional community based study. BMC Womens Health.

[11] Pirkle CM, de Albuquerque Sousa AC, Alvarado B, Zunzunegui MV (2014). Early maternal age at first birth is associated with chronic diseases and poor physical performance in older age: cross-sectional analysis from the International Mobility in Aging Study. BMC Public Health.

[12] Wu P, Mamas MA, Gulati M (2019). Pregnancy As a Predictor of Maternal Cardiovascular Disease: The Era of CardioObstetrics. J Womens Health (Larchmt).

[13] Williams D (2003). Pregnancy: a stress test for life. Curr Opin Obstet Gynecol.

[14] Paauw ND, van Rijn BB, Lely AT, Joles JA (2017). Pregnancy as a critical window for blood pressure regulation in mother and child: programming and reprogramming. Acta Physiol (Oxf).

[15] Guarrera S, Fiorito G, Onland-Moret NC, Russo A, Agnoli C, Allione A, Di Gaetano C, Mattiello A, Ricceri F, Chiodini P (2015). Gene-specific DNA methylation profiles and LINE-1 hypomethylation are associated with myocardial infarction risk. Clin Epigenet.

[16] Gomez-Alonso MDC, Kretschmer A, Wilson R, Pfeiffer L, Karhunen V, Seppälä I, Zhang W, Mittelstraß K, Wahl S, Matias-Garcia PR (2021). DNA methylation and lipid metabolism: an EWAS of 226 metabolic measures. Clin Epigenet.

[17] Portilla-Fernández E, Hwang SJ, Wilson R, Maddock J, Hill WD, Teumer A, Mishra PP, Brody JA, Joehanes R, Ligthart S et al: Meta-analysis of epigenome-wide association studies of carotid intima-media thickness. Eur J Epidemiol 2021. 10.1007/s10654-021-00759-z PMC8629903 34091768

[18] Horvath S, Gurven M, Levine ME, Trumble BC, Kaplan H, Allayee H, Ritz BR, Chen B, Lu AT, Rickabaugh TM (2016). An epigenetic clock analysis of race/ethnicity, sex, and coronary heart disease. Genome Biol.

[19] Chen BH, Marioni RE, Colicino E, Peters MJ, Ward-Caviness CK, Tsai PC, Roetker NS, Just AC, Demerath EW, Guan W (2016). DNA methylation-based measures of biological age: meta-analysis predicting time to death. Aging (Albany NY).

[20] Ryan CP, Hayes MG, Lee NR, McDade TW, Jones MJ, Kobor MS, Kuzawa CW, Eisenberg DTA (2018). Reproduction predicts shorter telomeres and epigenetic age acceleration among young adult women. Sci Rep.

[21] Kresovich JK, Harmon QE, Xu Z, Nichols HB, Sandler DP, Taylor JA (2019). Reproduction, DNA methylation and biological age. Hum Reprod.

[22] Ross KM, Carroll J, Horvath S, Hobel CJ, Coussons-Read ME, Dunkel Schetter C (2020). Immune epigenetic age in pregnancy and 1 year after birth: Associations with weight change. Am J Reprod Immunol.

[23] Ross KM, Carroll JE, Horvath S, Hobel CJ, Coussons-Read ME, Dunkel Schetter C (2020). Epigenetic age and pregnancy outcomes: GrimAge acceleration is associated with shorter gestational length and lower birthweight. Clin Epigenet.

[24] Agha G, Hajj H, Rifas-Shiman SL, Just AC, Hivert MF, Burris HH, Lin X, Litonjua AA, Oken E, DeMeo DL (2016). Birth weight-for-gestational age is associated with DNA methylation at birth and in childhood. Clin Epigenet.

[25] Raitakari OT, Juonala M, Ronnemaa T, Keltikangas-Jarvinen L, Rasanen L, Pietikainen M, Hutri-Kahonen N, Taittonen L, Jokinen E, Marniemi J (2008). Cohort profile: The Cardiovascular Risk in Young Finns Study. Int J Epidemiol.

[26] Triche TJ, Weisenberger DJ, Van Den Berg D, Laird PW, Siegmund KD (2013). Low-level processing of Illumina Infinium DNA Methylation BeadArrays. Nucleic Acids Res.

[27] Aryee MJ, Jaffe AE, Corrada-Bravo H, Ladd-Acosta C, Feinberg AP, Hansen KD, Irizarry RA (2014). Minfi: a flexible and comprehensive Bioconductor package for the analysis of Infinium DNA methylation microarrays. Bioinformatics.

[28] Lehne B, Drong AW, Loh M, Zhang W, Scott WR, Tan ST, Afzal U, Scott J, Jarvelin MR, Elliott P (2015). A coherent approach for analysis of the Illumina HumanMethylation450 BeadChip improves data quality and performance in epigenome-wide association studies. Genome Biol.

[29] McCartney DL, Walker RM, Morris SW, McIntosh AM, Porteous DJ, Evans KL (2016). Identification of polymorphic and off-target probe binding sites on the Illumina Infinium MethylationEPIC BeadChip. Genom Data.

[30] Pidsley R, Zotenko E, Peters TJ, Lawrence MG, Risbridger GP, Molloy P, Van Djik S, Muhlhausler B, Stirzaker C, Clark SJ (2016). Critical evaluation of the Illumina MethylationEPIC BeadChip microarray for whole-genome DNA methylation profiling. Genome Biol.

[31] R: A language and environment for statistical computing https://www.R-project.org/

[32] Houseman EA, Accomando WP, Koestler DC, Christensen BC, Marsit CJ, Nelson HH, Wiencke JK, Kelsey KT (2012). DNA methylation arrays as surrogate measures of cell mixture distribution. BMC Bioinf.

[33] Phipson B, Maksimovic J, Oshlack A (2016). missMethyl: an R package for analyzing data from Illumina's HumanMethylation450 platform. Bioinformatics.

[34] Hannum G, Guinney J, Zhao L, Zhang L, Hughes G, Sadda S, Klotzle B, Bibikova M, Fan JB, Gao Y (2013). Genome-wide methylation profiles reveal quantitative views of human aging rates. Mol Cell.

[35] Levine ME, Lu AT, Quach A, Chen BH, Assimes TL, Bandinelli S, Hou L, Baccarelli AA, Stewart JD, Li Y (2018). An epigenetic biomarker of aging for lifespan and healthspan. Aging.

[36] Lu AT, Quach A, Wilson JG, Reiner AP, Aviv A, Raj K, Hou L, Baccarelli AA, Li Y, Stewart JD (2019). DNA methylation GrimAge strongly predicts lifespan and healthspan. Aging.

[37] Hutcheon JA, Lisonkova S, Joseph KS (2011). Epidemiology of pre-eclampsia and the other hypertensive disorders of pregnancy. Best Pract Res Clin Obstet Gynaecol.

[38] Huang Y, Ollikainen M, Muniandy M, Zhang T, van Dongen J, Hao G, van der Most PJ, Pan Y, Pervjakova N, Sun YV (2020). Identification, heritability, and relation with gene expression of novel dna methylation loci for blood pressure. Hypertension.

[39] Mishra PP, Hänninen I, Raitoharju E, Marttila S, Mishra BH, Mononen N, Kähönen M, Hurme M, Raitakari O, Törönen P et al: Epigenome-450K-wide methylation signatures of active cigarette smoking: The Young Finns Study. Biosci Rep 2020, 40(7). 10.1042/BSR20200596 PMC7340865 32583859

[40] Engin A (2017). Adiponectin-resistance in obesity. Adv Exp Med Biol.

[41] Wang B, Lin H, Li X, Lu W, Kim JB, Xu A, Cheng KKY (2020). The adaptor protein APPL2 controls glucose-stimulated insulin secretion via F-actin remodeling in pancreatic β-cells. Proc Natl Acad Sci USA.

[42] Arai E, Gotoh M, Tian Y, Sakamoto H, Ono M, Matsuda A, Takahashi Y, Miyata S, Totsuka H, Chiku S (2015). Alterations of the spindle checkpoint pathway in clinicopathologically aggressive CpG island methylator phenotype clear cell renal cell carcinomas. Int J Cancer.

[43] Chen J, Wu F, Shi Y, Yang D, Xu M, Lai Y, Liu Y (2019). Identification of key candidate genes involved in melanoma metastasis. Mol Med Rep.

[44] Fararjeh AS, Chen LC, Ho YS, Cheng TC, Liu YR, Chang HL, Chang HW, Wu CH, Tu SH: Proteasome 26S subunit, non-ATPase 3 (PSMD3) regulates breast cancer by stabilizing HER2 from degradation. Cancers 2019;11(4). 10.3390/cancers11040527 PMC6549480 31013812

[45] Yu B, Liang H, Ye Q, Wang Y: Establishment of a genomic-clinicopathologic nomogram for predicting early recurrence of hepatocellular carcinoma After R0 resection. J Gastrointest Surg 2020. 10.1007/s11605-020-04554-1 32128678

[46] Ching T, Ha J, Song MA, Tiirikainen M, Molnar J, Berry MJ, Towner D, Garmire LX (2015). Genome-scale hypomethylation in the cord blood DNAs associated with early onset preeclampsia. Clin Epigenetics.

[47] Stratton MS, Farina FM, Elia L (2019). Epigenetics and vascular diseases. J Mol Cell Cardiol.

[48] Tang Y, Liu H, Li H, Peng T, Gu W, Li X (2015). Hypermethylation of the HLA-G promoter is associated with preeclampsia. Mol Hum Reprod.

[49] Azizi F, Omrani MD, Amiri V, Mirfakhraie R, Dodangeh F, Shahmirzadi SA, Gargari SS (2019). Altered methylation and expression patterns of genes regulating placental nitric oxide pathway in patients with severe preeclampsia. Hum Antibodies.

[50] Ge J, Wang J, Zhang F, Diao B, Song ZF, Shan LL, Wang W, Cao HJ, Li XQ (2015). Correlation between MTHFR gene methylation and pre-eclampsia, and its clinical significance. Genet Mol Res.

[51] Oudejans C, Poutsma A, Michel O, Mulders J, Visser A, van Dijk M, Nauta T, Bokslag A, Paulus W, de Haas A (2016). Genome-wide identification of epigenetic hotspots potentially related to cardiovascular risk in adult women after a complicated pregnancy. PLoS ONE.

[52] Ariff A, Melton PE, Brennecke SP, Moses EK (2019). Analysis of the epigenome in multiplex pre-eclampsia families identifies SORD, DGKI, and ICA1 as novel candidate risk genes. Front Genet.

[53] Enquobahrie DA, Moore A, Muhie S, Tadesse MG, Lin S, Williams MA (2015). Early pregnancy maternal blood DNA methylation in repeat pregnancies and change in gestational diabetes mellitus status—a pilot study. Reprod Sci.

[54] Kang J, Lee CN, Li HY, Hsu KH, Lin SY (2017). Genome-wide DNA methylation variation in maternal and cord blood of gestational diabetes population. Diabetes Res Clin Pract.

[55] Wu P, Farrell WE, Haworth KE, Emes RD, Kitchen MO, Glossop JR, Hanna FW, Fryer AA (2018). Maternal genome-wide DNA methylation profiling in gestational diabetes shows distinctive disease-associated changes relative to matched healthy pregnancies. Epigenetics.

[56] Kang J, Lee CN, Li HY, Hsu KH, Wang SH, Lin SY (2018). Association of interleukin-10 methylation levels with gestational diabetes in a Taiwanese population. Front Genet.

[57] Li E, Luo T, Wang Y (2019). Identification of diagnostic biomarkers in patients with gestational diabetes mellitus based on transcriptome gene expression and methylation correlation analysis. Reprod Biol Endocrinol.

[58] Dias S, Adam S, Rheeder P, Louw J, Pheiffer C: Altered genome-wide DNA methylation in peripheral blood of South African women with gestational diabetes mellitus. Int J Mol Sci 2019, 20(23). 10.3390/ijms20235828 PMC6928622 31757015

[59] White WM, Brost BC, Sun Z, Rose C, Craici I, Wagner SJ, Turner S, Garovic VD (2012). Normal early pregnancy: a transient state of epigenetic change favoring hypomethylation. Epigenetics.

[60] Wang T, Xiang Y, Zhou X, Zheng X, Zhang H, Zhang X, Zhang J, He L, Zhao X (2019). Epigenome-wide association data implicate fetal/maternal adaptations contributing to clinical outcomes in preeclampsia. Epigenomics.

[61] Li S, Kim E, Wong EM, Joo JE, Nguyen TL, Stone J, Song YM, Flander LB, Saffery R, Giles GG (2017). Twin birth changes DNA methylation of subsequent siblings. Sci Rep.

[62] Harville EW, Chen W, Guralnik J, Bazzano LA (2018). Reproductive history and physical functioning in midlife: the Bogalusa Heart Study. Maturitas.

[63] Xu Z, Niu L, Li L, Taylor JA (2016). ENmix: a novel background correction method for Illumina HumanMethylation450 BeadChip. Nucleic Acids Res.

