# Supplementary material for: Reproductive history and blood cell DNA methylation later in life: the Young Finns Study
Source: Clin Epigenetics. 2021 Dec 20;13:227. doi: 10.1186/s13148-021-01215-1 (PMC8690999; doi:10.1186/s13148-021-01215-1)

| Table S1. CpG sites with differential methylation in women with a history of pre-eclampsia. All sites meeting the genome-wide significance level. Fourteen CpG sites met the genome-wide significance criterion (p<5 x 10^-8^) for both pre-eclampsia and gestational hypertension (starred*). | | | | | |
| --- | --- | --- | --- | --- | --- |
| CpG | Beta | SE | p-value | chromosome | Gencode basic name |
| cg16202187* | 0.02063 | 0.00191 | 5.75E-25 | chr19 | HAUS8 |
| cg05583353* | -0.04759 | 0.00466 | 1.02E-22 | chr2 | AC092652.1 |
| cg10918328* | 0.04075 | 0.00410 | 1.16E-21 | chr13 | LINC00391 |
| cg26460602* | -0.05876 | 0.00647 | 1.39E-18 | chr19 |  |
| cg03942922 | -0.05361 | 0.00602 | 5.81E-18 | chr6 | C6orf130 |
| cg24936032 | 0.05858 | 0.00659 | 6.31E-18 | chr5 | HINT1 |
| cg24141991* | -0.04536 | 0.00516 | 1.48E-17 | chr11 | RNF26;RP11-334E6.10 |
| cg23567310 | -0.05816 | 0.00667 | 2.47E-17 | chr3 | YEATS2-AS1 |
| cg11835785 | -0.06245 | 0.00730 | 8.79E-17 | chr19 | KDM4B |
| cg08112740* | -0.07691 | 0.00902 | 1.13E-16 | chr19 |  |
| cg07904329 | 0.05445 | 0.00642 | 1.57E-16 | chr11 |  |
| cg14453326* | 0.04403 | 0.00520 | 1.89E-16 | chr12 | DDX55 |
| cg13709211* | -0.09271 | 0.01098 | 2.15E-16 | chr14 | MEG3 |
| cg19570702* | -0.06949 | 0.00823 | 2.23E-16 | chr6 | HCG25; VPS52 |
| cg25273619 | -0.04732 | 0.00562 | 2.45E-16 | chr13 | DCUN1D2-AS2 |
| cg05223360* | -0.05508 | 0.00655 | 2.77E-16 | chr1 |  |
| cg00263326 | -0.05014 | 0.00600 | 4.44E-16 | chr7 | AC007551.3 |
| ch.15.33220631R | 0.04902 | 0.00588 | 4.85E-16 | chr15 | RP11-323I15.5 |
| cg16505237 | 0.05600 | 0.00672 | 5.17E-16 | chr8 | DGAT1 |
| cg00926164 | -0.03864 | 0.00472 | 1.45E-15 | chr19 |  |
| cg26859716 | -0.08493 | 0.01044 | 2.28E-15 | chr6 |  |
| ch.1.179457656R | 0.05280 | 0.00651 | 2.72E-15 | chr1 |  |
| cg24133297 | -0.05808 | 0.00717 | 2.95E-15 | chr3 | TBC1D5 |
| cg05080811* | 0.05005 | 0.00619 | 3.24E-15 | chr22 |  |
| cg17324671* | -0.06305 | 0.00780 | 3.40E-15 | chr2 |  |
| ch.4.3207570F | 0.04603 | 0.00573 | 4.58E-15 | chr4 | RP11-287F9.2 |
| cg08751499 | 0.05516 | 0.00689 | 6.02E-15 | chr6 | EDN1 |
| cg01456695 | -0.05306 | 0.00664 | 6.41E-15 | chr11 | PTPRJ |
| cg10350607 | -0.05196 | 0.00653 | 8.73E-15 | chr8 |  |
| cg11853350 | -0.05247 | 0.00662 | 1.02E-14 | chr9 | RP11-548B3.3 |
| cg21642391 | -0.05402 | 0.00684 | 1.34E-14 | chr2 |  |
| cg23834489* | -0.04360 | 0.00559 | 2.60E-14 | chr17 | PSMD3 |
| cg20653654 | -0.04353 | 0.00562 | 3.83E-14 | chr4 | STIM2 |
| cg02428402 | -0.03222 | 0.00418 | 4.89E-14 | chr9 |  |
| cg24723798 | -0.04921 | 0.00643 | 7.56E-14 | chr2 |  |
| cg14916754 | -0.05162 | 0.00677 | 9.28E-14 | chr12 | EP400 |
| cg17048100 | 0.00915 | 0.00120 | 9.54E-14 | chr19 |  |
| cg11414540 | 0.04747 | 0.00624 | 1.08E-13 | chr19 | NR2C2AP |
| cg24555562 | -0.03178 | 0.00421 | 1.51E-13 | chr2 | AC131097.3 |
| cg00472766 | -0.04024 | 0.00533 | 1.56E-13 | chr1 | Y_RNA;WDR77 |
| cg23753247 | 0.01682 | 0.00225 | 2.66E-13 | chr11 | STK33 |
| cg21817450 | -0.04052 | 0.00552 | 6.68E-13 | chr13 | ENOX1 |
| cg15672329 | -0.04527 | 0.00619 | 8.12E-13 | chr11 | FCHSD2 |
| cg04943085 | 0.02488 | 0.00340 | 8.20E-13 | chr2 | RAB11FIP5 |
| cg22953445 | -0.04830 | 0.00661 | 8.27E-13 | chr6 | RFX6 |
| cg22988458 | -0.05111 | 0.00701 | 9.23E-13 | chr7 |  |
| cg22647092 | -0.05038 | 0.00692 | 1.00E-12 | chr7 | AC083884.8 |
| cg05360265 | 0.01909 | 0.00264 | 1.35E-12 | chr6 | STXBP5; RP11-497D6.4 |
| cg27654083* | -0.04766 | 0.00659 | 1.42E-12 | chr14 |  |
| cg13432087 | -0.04724 | 0.00656 | 1.76E-12 | chr8 | ANK1 |
| cg12600163 | 0.02011 | 0.00280 | 2.02E-12 | chr20 | NAA20 |
| cg00933692 | -0.04906 | 0.00684 | 2.05E-12 | chr17 | MYH1 |
| cg06258294 | -0.05517 | 0.00770 | 2.23E-12 | chr8 |  |
| cg09945592 | 0.01946 | 0.00272 | 2.39E-12 | chr10 | RP11-57C13.6;RP11-57C13.3 |
| cg01099150 | -0.05591 | 0.00782 | 2.46E-12 | chr2 | AC011747.6 |
| cg18240347 | -0.04998 | 0.00701 | 2.86E-12 | chr17 | LRRC48 |
| cg27227281 | -0.04607 | 0.00659 | 6.83E-12 | chr7 |  |
| cg16243665 | 0.01194 | 0.00171 | 8.38E-12 | chr19 | LONP1; CATSPERD |
| cg06645624 | 0.03494 | 0.00502 | 8.63E-12 | chr8 | PTK2 |
| cg15654661 | -0.02685 | 0.00386 | 9.11E-12 | chr3 |  |
| cg21775370 | -0.04477 | 0.00646 | 1.07E-11 | chr20 | RIN2 |
| cg21990063 | 0.02255 | 0.00327 | 1.28E-11 | chr14 | ATP5S;L2HGDH |
| cg18568067 | -0.07350 | 0.01069 | 1.51E-11 | chr11 | DGKZ |
| cg00399614 | -0.08658 | 0.01262 | 1.65E-11 | chr5 |  |
| cg14334041 | -0.05069 | 0.00743 | 2.17E-11 | chr9 |  |
| cg02846312 | -0.01884 | 0.00277 | 2.50E-11 | chr16 |  |
| cg08923680 | 0.02478 | 0.00366 | 2.92E-11 | chr3 | RP11-520A21.1 |
| cg24593363 | -0.04593 | 0.00678 | 2.96E-11 | chr1 | RP3-395M20.8 |
| cg18550517 | -0.04772 | 0.00707 | 3.46E-11 | chr19 | VAV1 |
| cg10194503 | -0.04367 | 0.00649 | 3.82E-11 | chr1 |  |
| cg24931556 | -0.04809 | 0.00714 | 3.83E-11 | chr1 | RP11-328D5.1 |
| cg08978399 | -0.01601 | 0.00238 | 3.87E-11 | chr3 |  |
| cg07441964 | 0.01960 | 0.00292 | 4.48E-11 | chr11 | FTH1 |
| cg00773459 | 0.01962 | 0.00293 | 5.06E-11 | chr1 | TTLL7 |
| cg02781074 | -0.07433 | 0.01112 | 5.21E-11 | chr13 | A2LD1 |
| cg02567323 | -0.05031 | 0.00755 | 6.08E-11 | chr2 |  |
| cg01758807 | -0.04793 | 0.00720 | 6.20E-11 | chr7 |  |
| cg13448512 | -0.05078 | 0.00764 | 6.60E-11 | chr8 |  |
| cg05620980 | -0.07316 | 0.01105 | 7.72E-11 | chr7 | AC147651.3 |
| cg10907237 | 0.05230 | 0.00791 | 8.00E-11 | chr13 | LINC00391 |
| cg23662138 | 0.07775 | 0.01180 | 9.32E-11 | chr21 | ADARB1 |
| cg14154527 | -0.04355 | 0.00661 | 9.53E-11 | chr7 | SDK1 |
| cg09127764 | -0.06261 | 0.00952 | 1.02E-10 | chr6 | RP11-497D6.4 |
| cg02492912 | -0.03950 | 0.00601 | 1.04E-10 | chr2 |  |
| cg11728484 | 0.01735 | 0.00264 | 1.07E-10 | chr9 | CKS2 |
| cg23380216 | 0.02250 | 0.00344 | 1.23E-10 | chr12 | POP5 |
| cg16736242 | 0.01658 | 0.00254 | 1.33E-10 | chr8 | FDFT1;RP11-297N6.4 |
| cg01206691 | 0.00997 | 0.00153 | 1.39E-10 | chr8 | ZNF623 |
| cg23799162 | -0.04264 | 0.00654 | 1.42E-10 | chr6 | RP11-524C21.2 |
| cg11884093 | 0.01290 | 0.00199 | 1.81E-10 | chr19 | PIN1 |
| cg22593746 | -0.05165 | 0.00797 | 1.84E-10 | chr20 |  |
| cg15904054 | -0.04822 | 0.00745 | 1.93E-10 | chr14 |  |
| cg12522736 | 0.01156 | 0.00179 | 2.04E-10 | chr19 | UBE2M |
| cg11400143 | -0.04805 | 0.00744 | 2.16E-10 | chr6 |  |
| cg12495889 | -0.05429 | 0.00844 | 2.51E-10 | chr22 |  |
| cg05365469 | -0.02770 | 0.00432 | 2.75E-10 | chr1 | UCHL5 |
| cg15586755 | -0.07201 | 0.01125 | 3.05E-10 | chrX | ATP11C |
| cg23542284 | 0.02404 | 0.00376 | 3.11E-10 | chr19 | NR2C2AP |
| cg22754309 | -0.01940 | 0.00304 | 3.29E-10 | chr6 |  |
| cg20712058 | 0.02648 | 0.00419 | 5.17E-10 | chr3 | CHST13 |
| cg07021538 | 0.04414 | 0.00700 | 5.38E-10 | chr6 | PPP1R11 |
| cg10096055 | 0.01791 | 0.00284 | 5.48E-10 | chr1 | TMEM52 |
| cg16823617 | -0.01935 | 0.00307 | 5.69E-10 | chr9 | DENND1A |
| cg12313606 | -0.05444 | 0.00866 | 6.11E-10 | chr4 |  |
| cg13687158 | -0.02551 | 0.00407 | 6.57E-10 | chr15 | SNRPN |
| cg02833117 | -0.01898 | 0.00303 | 6.74E-10 | chr2 | ERMN |
| cg04159666 | -0.02024 | 0.00323 | 7.00E-10 | chr6 | RP3-325F22.3;RN5S219;MAP3K5 |
| cg03703171 | -0.04074 | 0.00651 | 7.23E-10 | chr2 | IL1RN |
| cg02283796 | -0.02129 | 0.00340 | 7.31E-10 | chr19 | AURKC |
| cg09618514 | -0.04954 | 0.00794 | 8.05E-10 | chr21 | SLC37A1 |
| cg10362320 | 0.05716 | 0.00916 | 8.18E-10 | chr17 | CYB5D2;ZZEF1 |
| cg03705875 | -0.04332 | 0.00696 | 8.72E-10 | chr14 | CTD-2207P18.1;LTBP2 |
| cg05917225 | 0.04257 | 0.00684 | 8.76E-10 | chr6 | NEDD9; RP3-510L9.1 |
| cg03114560 | -0.02452 | 0.00395 | 9.93E-10 | chr13 |  |
| cg05691168 | 0.04119 | 0.00664 | 1.02E-09 | chr2 | ATIC |
| cg09837632 | 0.03998 | 0.00646 | 1.08E-09 | chr2 | ATIC |
| cg12731137 | 0.01494 | 0.00242 | 1.18E-09 | chr17 | ALOXE3 |
| cg03462828 | -0.10322 | 0.01672 | 1.22E-09 | chr11 | TMEM138 |
| cg02686200 | -0.04377 | 0.00710 | 1.25E-09 | chr17 |  |
| cg05998153 | 0.02046 | 0.00332 | 1.26E-09 | chr5 |  |
| cg09847139 | -0.05195 | 0.00843 | 1.31E-09 | chr7 |  |
| cg25191519 | -0.04061 | 0.00660 | 1.36E-09 | chr16 | U7;SPG7 |
| cg06934478 | -0.04617 | 0.00752 | 1.45E-09 | chr20 |  |
| cg18583248 | -0.05188 | 0.00847 | 1.60E-09 | chr3 |  |
| cg07221298 | -0.07354 | 0.01202 | 1.69E-09 | chr16 |  |
| cg20728685 | 0.01884 | 0.00308 | 1.73E-09 | chr5 | GPBP1 |
| cg10123842 | -0.05179 | 0.00849 | 1.88E-09 | chr3 |  |
| cg15453315 | -0.05399 | 0.00886 | 1.91E-09 | chr17 | SPNS3 |
| cg07058528 | 0.01849 | 0.00304 | 1.96E-09 | chr17 | C17orf49 |
| cg06007456 | -0.03348 | 0.00550 | 2.04E-09 | chr7 | XRCC2 |
| cg25835515 | -0.02924 | 0.00482 | 2.28E-09 | chr21 |  |
| cg14245202 | -0.01485 | 0.00246 | 2.68E-09 | chr17 | AC087645.1 |
| cg05025179 | 0.01465 | 0.00243 | 2.86E-09 | chr6 | C6orf136 |
| cg24491776 | -0.04759 | 0.00790 | 2.88E-09 | chr6 | RP3-468B3.3 |
| cg01891969 | -0.03540 | 0.00588 | 2.93E-09 | chr13 |  |
| cg08264907 | -0.02376 | 0.00395 | 3.13E-09 | chr4 |  |
| cg12867142 | -0.04965 | 0.00827 | 3.24E-09 | chr4 | TSPAN5 |
| cg24875440 | 0.01809 | 0.00302 | 3.38E-09 | chr1 |  |
| cg11196544 | -0.04252 | 0.00709 | 3.39E-09 | chr12 |  |
| cg10845251 | 0.02568 | 0.00429 | 3.77E-09 | chr19 | NR2C2AP |
| cg20999864 | -0.03451 | 0.00579 | 4.31E-09 | chr7 | HNRNPA2B1 |
| cg13138226 | -0.04452 | 0.00748 | 4.53E-09 | chr12 |  |
| cg07414863 | -0.03862 | 0.00651 | 4.84E-09 | chr10 |  |
| cg04636885 | -0.09783 | 0.01648 | 4.89E-09 | chr4 |  |
| cg23889338 | 0.04193 | 0.00711 | 6.05E-09 | chr19 | DNAJB1 |
| cg14813294 | -0.03336 | 0.00566 | 6.07E-09 | chr12 | C12orf69 |
| cg05788548 | -0.04498 | 0.00763 | 6.19E-09 | chr1 | SKI |
| cg16725225 | -0.02206 | 0.00374 | 6.20E-09 | chr8 |  |
| cg23249667 | 0.03458 | 0.00588 | 6.54E-09 | chr6 | ATP6V1G2-DDX39B;NFKBIL1 |
| cg05865860 | 0.01101 | 0.00187 | 6.57E-09 | chr7 | WDR60 |
| cg13033090 | 0.02585 | 0.00441 | 7.21E-09 | chr18 | CELF4 |
| cg24215868 | -0.04750 | 0.00810 | 7.22E-09 | chr21 |  |
| cg10900351 | -0.04073 | 0.00696 | 7.80E-09 | chr12 |  |
| cg07036412 | 0.02220 | 0.00379 | 7.85E-09 | chr6 |  |
| cg17633015 | -0.06277 | 0.01073 | 7.98E-09 | chr7 | MAD1L1 |
| cg11767185 | -0.04971 | 0.00851 | 8.24E-09 | chrX | DIAPH2-AS1 |
| cg16998962 | 0.01121 | 0.00193 | 9.29E-09 | chr2 | SLC30A6 |
| cg07530460 | -0.04387 | 0.00757 | 1.07E-08 | chr17 | U6 |
| cg06038495 | -0.04388 | 0.00757 | 1.07E-08 | chr15 | UBE3A; SNHG14 |
| cg23672425 | -0.01688 | 0.00291 | 1.12E-08 | chr1 | ANGPTL7 |
| cg09941017 | -0.01047 | 0.00181 | 1.18E-08 | chr7 |  |
| cg05907238 | -0.05538 | 0.00959 | 1.23E-08 | chr11 | PIWIL4; RP11-867G2.8 |
| cg01109734 | -0.03987 | 0.00692 | 1.31E-08 | chr2 | AC113607.1;AC113607.1 |
| cg08931968 | -0.04591 | 0.00797 | 1.31E-08 | chr6 | ZNRD1-AS1 |
| cg26837800 | 0.03774 | 0.00656 | 1.38E-08 | chr1 |  |
| cg09827858 | -0.01334 | 0.00232 | 1.39E-08 | chr1 | TMEM59 |
| cg27613592 | -0.03270 | 0.00569 | 1.40E-08 | chr1 | PTPRF |
| cg06760466 | -0.02451 | 0.00426 | 1.41E-08 | chr2 |  |
| cg15893895 | -0.02301 | 0.00400 | 1.41E-08 | chr7 | COBL |
| cg15694879 | 0.01225 | 0.00214 | 1.53E-08 | chr1 | HEATR8 |
| cg06113755 | 0.04698 | 0.00820 | 1.60E-08 | chr1 | EYA3 |
| cg25389462 | 0.02001 | 0.00350 | 1.73E-08 | chr1 | OPN3 |
| cg24933241 | -0.03902 | 0.00683 | 1.74E-08 | chr1 |  |
| cg03293513 | 0.04928 | 0.00864 | 1.82E-08 | chr16 | NME3 |
| cg12276123 | 0.04115 | 0.00722 | 1.87E-08 | chr2 | AC061961.2;KCNJ3 |
| cg05729047 | -0.05108 | 0.00896 | 1.87E-08 | chr1 |  |
| cg10845232 | -0.05308 | 0.00933 | 1.94E-08 | chr6 |  |
| cg05022385 | -0.04420 | 0.00777 | 1.96E-08 | chr5 | RAI14 |
| cg10087787 | -0.04342 | 0.00764 | 2.01E-08 | chr1 |  |
| cg11474532 | 0.01235 | 0.00217 | 2.05E-08 | chr5 | DEPDC1B |
| cg26338791 | 0.02471 | 0.00436 | 2.16E-08 | chr22 | RP11-398F12.1 |
| cg11267619 | 0.01948 | 0.00344 | 2.24E-08 | chr7 | AC007255.8;PRR15 |
| cg17309380 | -0.07314 | 0.01293 | 2.34E-08 | chr14 | SYNE2 |
| cg24760819 | -0.04928 | 0.00872 | 2.46E-08 | chr14 |  |
| cg17314277 | -0.06298 | 0.01117 | 2.60E-08 | chr1 | NMNAT2 |
| cg16750845 | 0.00678 | 0.00120 | 2.64E-08 | chr14 | NEMF |
| ch.12.292758R | 0.00991 | 0.00176 | 2.65E-08 | chr12 | CSDA |
| cg03984604 | -0.05309 | 0.00944 | 2.83E-08 | chr1 |  |
| cg03603214 | 0.01898 | 0.00337 | 2.83E-08 | chr1 | BEND5;BEND5 |
| cg00136822 | -0.02765 | 0.00492 | 2.84E-08 | chr12 | SLC38A1 |
| cg05420621 | -0.04267 | 0.00761 | 3.16E-08 | chr20 |  |
| cg03265533 | -0.03016 | 0.00539 | 3.28E-08 | chr4 |  |
| cg11472064 | 0.01551 | 0.00277 | 3.33E-08 | chr1 | NCDN;KIAA0319L |
| cg03956606 | -0.02469 | 0.00443 | 3.63E-08 | chr1 | ANGPTL7 |
| cg21541159 | -0.04277 | 0.00767 | 3.65E-08 | chr2 |  |
| cg20846311 | -0.07731 | 0.01387 | 3.70E-08 | chr13 | NAA16 |
| cg23371584 | 0.19689 | 0.03536 | 3.85E-08 | chr1 | RP11-134P9.1 |
| cg01491360 | -0.06667 | 0.01200 | 4.06E-08 | chr19 |  |
| cg02879917 | 0.01447 | 0.00260 | 4.11E-08 | chr12 | FAM216A;GPN3 |
| cg00874019 | -0.02448 | 0.00441 | 4.28E-08 | chr7 | ACTR3B |
| cg03755333 | -0.03108 | 0.00561 | 4.45E-08 | chr19 | GATAD2A |
| cg09024381 | -0.03266 | 0.00590 | 4.53E-08 | chr5 |  |
| cg26423160 | -0.02735 | 0.00494 | 4.57E-08 | chr7 |  |
| cg12582819 | -0.01424 | 0.00257 | 4.60E-08 | chr18 | TMEM241 |
| cg15315325 | -0.05231 | 0.00947 | 4.94E-08 | chr20 | ZMYND8 |
| cg16689534 | -0.04256 | 0.00771 | 4.98E-08 | chr9 | TRAF1 |

Table S2. CpG sites differentially methylated in women with a history of hypertensive disorders of pregnancy. CpG sites that met the genome-wide significance criterion (p<5 x 10^-8^) for both pre-eclampsia and gestational hypertension (starred*).

| CpG | Beta | SE | p value | chr | Gencode Basic |
| --- | --- | --- | --- | --- | --- |
| cg20089264 | 0.01708 | 0.00257 | 6.25E-11 | chr22 | ASCC2 |
| cg16202187* | 0.00891 | 0.00137 | 1.68E-10 | chr19 | HAUS8 |
| cg14862827 | 0.01052 | 0.00162 | 1.79E-10 | chr9 |  |
| cg09020375 | -0.0381 | 0.00609 | 7.65E-10 | chr4 | ART3 |
| cg23694630 | -0.0391 | 0.00629 | 9.20E-10 | chr10 |  |
| cg13081185 | -0.0341 | 0.00556 | 1.60E-09 | chr11 | SIK3 |
| cg12522722 | -0.0468 | 0.00765 | 1.63E-09 | chr11 | ZW10;RP11-661I21.2 |
| cg10918328* | 0.01762 | 0.00292 | 2.64E-09 | chr13 | LINC00391 |
| cg02068524 | 0.02589 | 0.00429 | 2.79E-09 | chr4 |  |
| cg08687948 | -0.0274 | 0.00456 | 3.09E-09 | chr7 | CYTH3;CYTH3 |
| cg06853492 | -0.0252 | 0.00426 | 5.69E-09 | chr2 | RAPGEF4 |
| cg17324671* | -0.032 | 0.00542 | 5.92E-09 | chr2 |  |
| cg26460602* | -0.0268 | 0.00455 | 6.75E-09 | chr19 |  |
| cg05223360* | -0.0269 | 0.00457 | 6.86E-09 | chr1 |  |
| cg15400652 | -0.0194 | 0.00331 | 8.20E-09 | chr19 | PSG2 |
| cg09138430 | 0.01593 | 0.00275 | 1.04E-08 | chr7 | DBNL |
| cg23834489* | -0.0225 | 0.00387 | 1.04E-08 | chr17 | PSMD3 |
| cg04806794 | 0.02776 | 0.00479 | 1.11E-08 | chr9 | TLE4 |
| cg18304195 | 0.02559 | 0.00445 | 1.40E-08 | chr3 | KBTBD8 |
| cg13709211* | -0.0437 | 0.00768 | 1.92E-08 | chr14 | MEG3 |
|  |  |  |  |  |  |
| cg14453326* | 0.02056 | 0.00364 | 2.51E-08 | chr12 | DDX55 |
| cg05583353* | -0.0188 | 0.00334 | 2.78E-08 | chr2 | AC092652.1 |
| cg13996392 | 0.02525 | 0.0045 | 3.06E-08 | chr4 | NEIL3 |
| cg08112740* | -0.0354 | 0.00632 | 3.29E-08 | chr19 |  |
| cg24141991* | -0.0203 | 0.00363 | 3.37E-08 | chr11 | RNF26;RP11-334E6.10 |
| cg17699666 | -0.0204 | 0.00366 | 3.46E-08 | chr2 | RFTN2 |
| cg05080811* | 0.02406 | 0.00431 | 3.68E-08 | chr22 |  |
| cg16112157 | -0.0283 | 0.00507 | 3.73E-08 | chr12 | KRT6A |
| cg19570702* | -0.0321 | 0.00576 | 3.81E-08 | chr6 | HCG25VPS52;VPS52 |
| cg03824048 | -0.0333 | 0.00598 | 3.95E-08 | chr9 |  |
| cg27654083* | -0.0252 | 0.00455 | 4.52E-08 | chr14 |  |

| Table S3. Top biological pathways associated with PE and PIH, by the KEGG and GO methods. None met the criteria for even nominal significance | | | | | | |  |
| --- | --- | --- | --- | --- | --- | --- | --- |
| PIH |  |  |  |  |  |  | |
|  | ONTOLOGY | TERM | N | DE | P.DE | FDR | |
| GO |  |  |  |  |  |  | |
| GO:0001897 | BP | cytolysis by symbiont of host cells | 1 | 1 | 0.001 | 1.000 | |
| GO:0001898 | BP | regulation of cytolysis by symbiont of host cells | 1 | 1 | 0.001 | 1.000 | |
| GO:0001899 | BP | negative regulation of cytolysis by symbiont of host cells | 1 | 1 | 0.001 | 1.000 | |
| GO:0051711 | BP | negative regulation of killing of cells of other organism | 1 | 1 | 0.001 | 1.000 | |
| GO:0051713 | BP | negative regulation of cytolysis in other organism | 1 | 1 | 0.001 | 1.000 | |
| GO:0051802 | BP | regulation of cytolysis in other organism involved in symbiotic interaction | 1 | 1 | 0.001 | 1.000 | |
| GO:0051803 | BP | negative regulation of cytolysis in other organism involved in symbiotic interaction | 1 | 1 | 0.001 | 1.000 | |
| GO:2000536 | BP | negative regulation of entry of bacterium into host cell | 2 | 1 | 0.002 | 1.000 | |
| GO:0099023 | CC | tethering complex | 65 | 2 | 0.002 | 1.000 | |
| GO:0051801 | BP | cytolysis in other organism involved in symbiotic interaction | 2 | 1 | 0.002 | 1.000 | |
| GO:1905719 | BP | protein localization to perinuclear region of cytoplasm | 2 | 1 | 0.002 | 1.000 | |
| GO:0099513 | CC | polymeric cytoskeletal fiber | 710 | 4 | 0.002 | 1.000 | |
| GO:0099053 | CC | activating signal cointegrator 1 complex | 3 | 1 | 0.002 | 1.000 | |
| GO:0007439 | BP | ectodermal digestive tract development | 2 | 1 | 0.003 | 1.000 | |
| GO:0048611 | BP | embryonic ectodermal digestive tract development | 2 | 1 | 0.003 | 1.000 | |
| GO:1990423 | CC | RZZ complex | 3 | 1 | 0.003 | 1.000 | |
| GO:0070939 | CC | Dsl1/NZR complex | 3 | 1 | 0.003 | 1.000 | |
| GO:0051710 | BP | regulation of cytolysis in other organism | 4 | 1 | 0.003 | 1.000 | |
| GO:0018120 | BP | peptidyl-arginine ADP-ribosylation | 4 | 1 | 0.003 | 1.000 | |
| GO:0001907 | BP | killing by symbiont of host cells | 4 | 1 | 0.003 | 1.000 | |
|  |  |  |  |  |  |  | |
| KEGG |  |  |  |  |  |  | |
| path:hsa04072 | | Phospholipase D signaling pathway | 147 | 2 | 0.008 | 1.000 | |
| path:hsa03410 | | Base excision repair | 33 | 1 | 0.026 | 1.000 | |
| path:hsa04911 | | Insulin secretion | 86 | 1 | 0.077 | 1.000 | |
| path:hsa04670 | | Leukocyte transendothelial migration | 110 | 1 | 0.092 | 1.000 | |
| path:hsa04261 | | Adrenergic signaling in cardiomyocytes | 148 | 1 | 0.126 | 1.000 | |
| path:hsa05130 | | Pathogenic Escherichia coli infection | 193 | 1 | 0.157 | 1.000 | |
| path:hsa04015 | | Rap1 signaling pathway | 210 | 1 | 0.178 | 1.000 | |
| path:hsa04024 | | cAMP signaling pathway | 219 | 1 | 0.178 | 1.000 | |
| path:hsa05131 | | Shigellosis | 240 | 1 | 0.191 | 1.000 | |
| path:hsa05132 | | Salmonella infection | 247 | 1 | 0.197 | 1.000 | |
| path:hsa04144 | | Endocytosis | 247 | 1 | 0.207 | 1.000 | |
| path:hsa05010 | | Alzheimer disease | 366 | 0 | 1.000 | 1.000 | |
| path:hsa00010 | | Glycolysis / Gluconeogenesis | 66 | 0 | 1.000 | 1.000 | |
| path:hsa00020 | | Citrate cycle (TCA cycle) | 30 | 0 | 1.000 | 1.000 | |
| path:hsa00030 | | Pentose phosphate pathway | 29 | 0 | 1.000 | 1.000 | |
| path:hsa00040 | | Pentose and glucuronate interconversions | 30 | 0 | 1.000 | 1.000 | |
| path:hsa00051 | | Fructose and mannose metabolism | 33 | 0 | 1.000 | 1.000 | |
| path:hsa00052 | | Galactose metabolism | 29 | 0 | 1.000 | 1.000 | |
| path:hsa00053 | | Ascorbate and aldarate metabolism | 27 | 0 | 1.000 | 1.000 | |
| path:hsa00061 | | Fatty acid biosynthesis | 17 | 0 | 1.000 | 1.000 | |
|  |  |  |  |  |  |  | |
| Pre-eclampsia | |  |  |  |  |  | |
| GO |  |  |  |  |  |  | |
| GO:0061051 | BP | positive regulation of cell growth involved in cardiac muscle cell development | 14 | 3 | 0.000 | 0.275 | |
| GO:0070603 | CC | SWI/SNF superfamily-type complex | 76 | 5 | 0.000 | 0.602 | |
| GO:1903691 | BP | positive regulation of wound healing, spreading of epidermal cells | 2 | 2 | 0.000 | 0.661 | |
| GO:0061050 | BP | regulation of cell growth involved in cardiac muscle cell development | 25 | 3 | 0.000 | 0.661 | |
| GO:1904949 | CC | ATPase complex | 101 | 5 | 0.000 | 0.768 | |
| GO:0097346 | CC | INO80-type complex | 25 | 3 | 0.000 | 0.838 | |
| GO:2000727 | BP | positive regulation of cardiac muscle cell differentiation | 29 | 3 | 0.000 | 1.000 | |
| GO:0099522 | CC | region of cytosol | 24 | 3 | 0.000 | 1.000 | |
| GO:0003298 | BP | physiological muscle hypertrophy | 38 | 3 | 0.001 | 1.000 | |
| GO:0003301 | BP | physiological cardiac muscle hypertrophy | 38 | 3 | 0.001 | 1.000 | |
| GO:0061049 | BP | cell growth involved in cardiac muscle cell development | 38 | 3 | 0.001 | 1.000 | |
| GO:0010613 | BP | positive regulation of cardiac muscle hypertrophy | 42 | 3 | 0.001 | 1.000 | |
| GO:0014742 | BP | positive regulation of muscle hypertrophy | 43 | 3 | 0.001 | 1.000 | |
| GO:1905209 | BP | positive regulation of cardiocyte differentiation | 41 | 3 | 0.001 | 1.000 | |
| GO:1903689 | BP | regulation of wound healing, spreading of epidermal cells | 6 | 2 | 0.001 | 1.000 | |
| GO:2000725 | BP | regulation of cardiac muscle cell differentiation | 50 | 3 | 0.001 | 1.000 | |
| GO:0055023 | BP | positive regulation of cardiac muscle tissue growth | 48 | 3 | 0.002 | 1.000 | |
| GO:0000812 | CC | Swr1 complex | 12 | 2 | 0.002 | 1.000 | |
| GO:1901216 | BP | positive regulation of neuron death | 94 | 4 | 0.002 | 1.000 | |
| GO:0031583 | BP | phospholipase D-activating G protein-coupled receptor signaling pathway | 1 | 1 | 0.002 | 1.000 | |
| KEGG |  |  |  |  |  |  | |
| path:hsa00561 | | Glycerolipid metabolism | 60 | 2 | 0.036 | 1.000 | |
| path:hsa05221 | | Acute myeloid leukemia | 67 | 2 | 0.060 | 1.000 | |
| path:hsa00100 | | Steroid biosynthesis | 20 | 1 | 0.080 | 1.000 | |
| path:hsa00670 | | One carbon pool by folate | 19 | 1 | 0.083 | 1.000 | |
| path:hsa05210 | | Colorectal cancer | 86 | 2 | 0.089 | 1.000 | |
| path:hsa05231 | | Choline metabolism in cancer | 97 | 2 | 0.110 | 1.000 | |
| path:hsa04066 | | HIF-1 signaling pathway | 108 | 2 | 0.116 | 1.000 | |
| path:hsa00532 | | Glycosaminoglycan biosynthesis - chondroitin sulfate / dermatan sulfate | 20 | 1 | 0.118 | 1.000 | |
| path:hsa04916 | | Melanogenesis | 101 | 2 | 0.121 | 1.000 | |
| path:hsa05215 | | Prostate cancer | 97 | 2 | 0.121 | 1.000 | |
| path:hsa01523 | | Antifolate resistance | 31 | 1 | 0.127 | 1.000 | |
| path:hsa05205 | | Proteoglycans in cancer | 204 | 3 | 0.128 | 1.000 | |
| path:hsa04136 | | Autophagy - other | 31 | 1 | 0.144 | 1.000 | |
| path:hsa04975 | | Fat digestion and absorption | 43 | 1 | 0.148 | 1.000 | |
| path:hsa04724 | | Glutamatergic synapse | 114 | 2 | 0.160 | 1.000 | |
| path:hsa03022 | | Basal transcription factors | 44 | 1 | 0.164 | 1.000 | |
| path:hsa00830 | | Retinol metabolism | 62 | 1 | 0.167 | 1.000 | |
| path:hsa04216 | | Ferroptosis | 41 | 1 | 0.176 | 1.000 | |
| path:hsa05017 | | Spinocerebellar ataxia | 141 | 2 | 0.179 | 1.000 | |
| path:hsa05216 | | Thyroid cancer | 37 | 1 | 0.200 | 1.000 | |

ID, Kyoto Encyclopedia of Genes and Genomes (KEGG) pathways ID

N, number of genes in the KEGG pathway

DE, number of genes that are differentially methylated

P.DE: p-value for over-representation of the KEGG pathway

FDR: false discovery rate

| Table S4. CpG sites associated with smoking and hypertension in this cohort in previous analyses | |
| --- | --- |
| Blood pressure[38] | Smoking[39] |
| cg19693031 | cg21408581 |
| cg01343041 | cg21566642 |
| cg19695041 | cg01940273 |
| cg13696706 | cg05951221 |
| cg11468085 | cg19859270 |
| cg00508575 | cg05575921 |
| cg05248321 | cg21161138 |
| cg02003183 | cg25648203 |
| cg12555233 | cg14817490 |
| cg07558761 | cg26703534 |
| cg07021906 | cg24090911 |
| cg04583842 | cg06126421 |
| cg08857797 | cg24859433 |
| cg02711608 | cg15342087 |
| cg06500161 | cg22851561 |
| cg01820192 | cg05284742 |
|  | cg03636183 |

Figure S1. Manhattan plots for parity (panel a) and preterm birth (panel b)


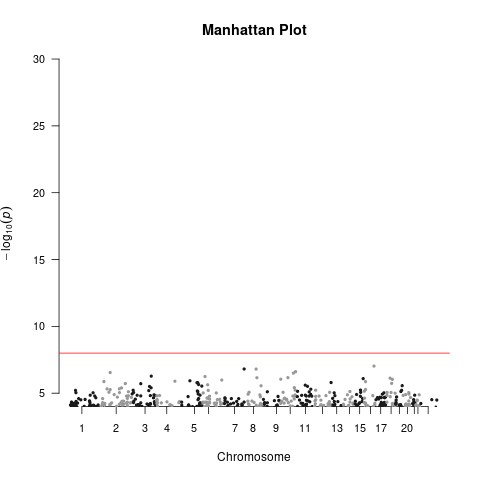

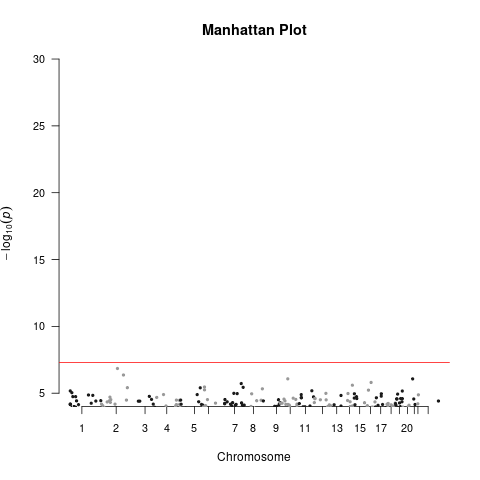


Figure S2. QQ plots for pre-eclampsia (panel 1) and hypertensive disorders (panel 2).


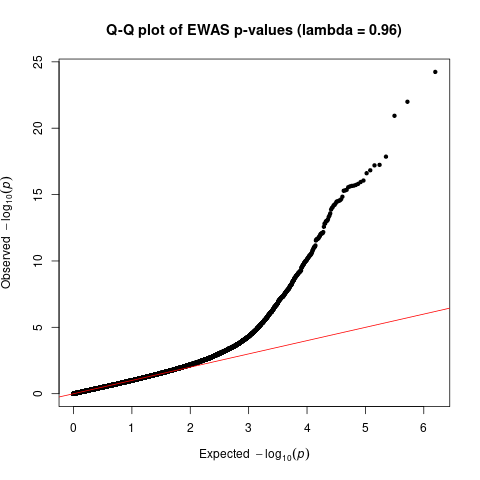

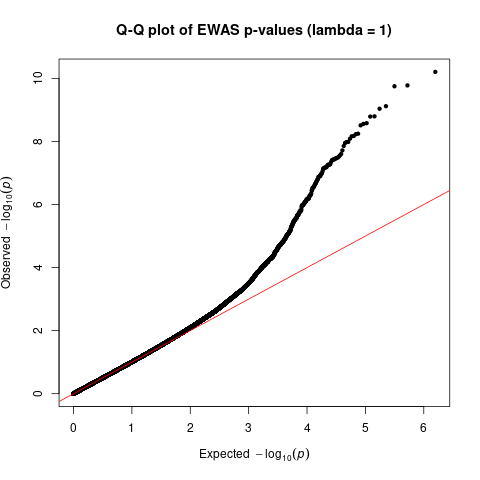

Supplement: Supplementary file 1 — Additional file 1. Supplementary tables and figures. [file 13148_2021_1215_MOESM1_ESM.docx]
